# Whole-exome sequencing identifies *SGCD* and *ACVRL1* mutations associated with total anomalous pulmonary venous return (TAPVR) in Chinese population

**DOI:** 10.18632/oncotarget.15434

**Published:** 2017-02-17

**Authors:** Jun Li, Shiwei Yang, Zhening Pu, Juncheng Dai, Tao Jiang, Fangzhi Du, Zhu Jiang, Yue Cheng, Genyin Dai, Jun Wang, Jirong Qi, Liming Cao, Xueying Cheng, Cong Ren, Xinli Li, Yuming Qin

**Affiliations:** ^1^ Department of Cardiology, Children's Hospital of Nanjing Medical University, Nanjing 210008, China; ^2^ Department of Epidemiology and Biostatistics, Jiangsu Key Lab of Cancer Biomarkers, Prevention and Treatment, Collaborative Innovation Center for Cancer Medicine, School of Public Health, Nanjing Medical University, Nanjing 211166, China; ^3^ Department of Cardiothoracic Surgery, Children's Hospital of Nanjing Medical University, Nanjing 210008, China; ^4^ Department of Cardiology, The First Affiliated Hospital, Nanjing Medical University, Nanjing 210029, China

**Keywords:** total anomalous pulmonary venous return (TAPVR), genetics, whole-exome sequencing (WES), rare genetic variant, congenital disease

## Abstract

As a rare type of Congenital Heart Defects (CHD), the genetic mechanism of Total Anomalous Pulmonary Venous Return (TAPVR) remains unknown, although previous studies have revealed potential disease-driving regions/genes. Blood samples collected from the 6 sporadic TAPVR cases and 81 non-TAPVR controls were subjected to whole exome sequencing. All detected variations were confirmed by direct Sanger sequencing. Here, we identified 2 non-synonymous missense mutations: c.C652T, p.R218W in activin A receptor type II-like 1 (ACVRL1), c.C717G, p.D239E in sarcoglycan delta (SGCD). Our results offered the landscape of mutations for TAPVR in Chinese population firstly and are valuable in the mutation-based pre- and post-natal screening and genetic diagnosis for TAPVR.

## INTRODUCTION

**Figure 1:**
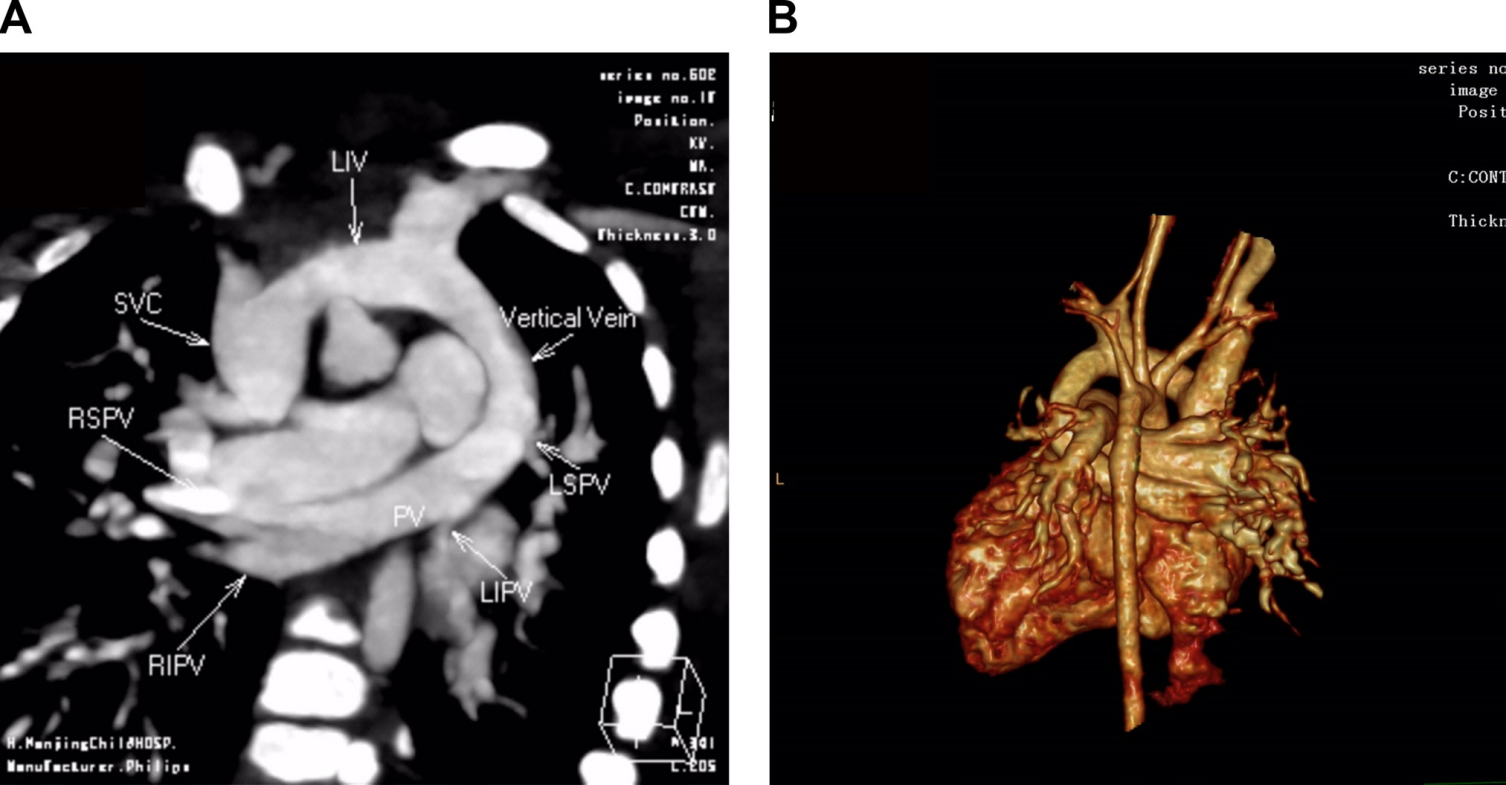
CT angiography in a 5-month-old girl with supracardiac total anomalous pulmonary venous connection (TAPVC) and unrestrictive ASD Multidetector CT angiography oblique coronal image (**A**) and volume rendered image (**B**) shows four individual pulmonary veins joining in a retrocardiac venous confluence and draining into the left innominate vein (LIV) via a vertical vein (VV). ASD: atrial septal defect; PV: pulmonary vein; SVC: superior vena cava; RIPV: right inferior pulmonary vein; RSPV: right superior pulmonary vein; LSPV: left superior pulmonary vein; LIPV: left inferior pulmonary vein; LIV: left innominate vein.

Total Anomalous Pulmonary Venous Return (TAPVR) (OMIM:%106700) is one type of cyanotic Congenital Heart Defects (CHD), in which none of the pulmonary veins connect to the left atrium and are malpositioned to the systemic venous circulation [1] (Figure 1A, 1B). TAPVR characterized by cardiac function deterioration including cyanosis, pulmonary hypertension, dyspnea, pulmonary edema, congestive heart failure affects 1 in 15,000 live births and 48.8% of them will die without surgery before the age of 1 [2]. Although TAPVR generally presents significant fatality rate, the pathogenesis is still vague and become hot research spot.

In 1960, Neill *et al*. described the first case that TAPVR in ‘scimitar syndrome’ [3]. Then, TAPVR has been associated with GATA binding protein 4 (*GATA4*), Zic family member 3 (*ZIC3*), and Gap junction protein and alpha 1 (*GJA1*) [4]. After a de novo 10;21 balanced translocation was reported, Cinquetti *et al*. defined *ANKRD1* as a possible candidate gene for TAPVR in 2008 [5]. Bleyl et al established a locus for TAPVR at 4p13-q12 [6] and found the *PDGFRA* as a driver gene in the further detailed research [7]. A non-synonymous variant in retinol binding protein 5 (RBP5) by Whole genome sequence (WGS) was analysised in 2 TAPVR patients recently [8]. Nevertheless, the contribution of known genes above was still limited to investigate the genetic cause. Therefore, we applied WES to the investigation of the genetic cause in 6 sporadic TAPVR cases and 81 non-TAPVR controls. We performed independent replication on additional 12 TAPVR patients by Sanger sequencing to identify the possible genetic variants.

## RESULTS

### Gene classification

After filtered through Public and in-house database, genes were classified referring to ACMG standards and guidelines [9] as follows: Category I genes were 15 TAPVR Pathogenic or likely Pathogenic Genes. (Supplementary Table 3); Category II genes were TAPVR associated Genes containing 221 human cardiac development related genes from gene ontology (GO) (Supplementary Table 4); Category III genes were unknown genes have not been reported previously. Correspondingly, Part I variants were in Category I genes. Part II variants were recurrent ones in Category II genes. Part III variants were not located in Category I genes and low frequency in the 1000 Genomes, Exome Sequencing Project (ESP) and the Exome Aggregation Consortium (ExAC, version 0.3).

### Genetic findings

**Figure 2:**
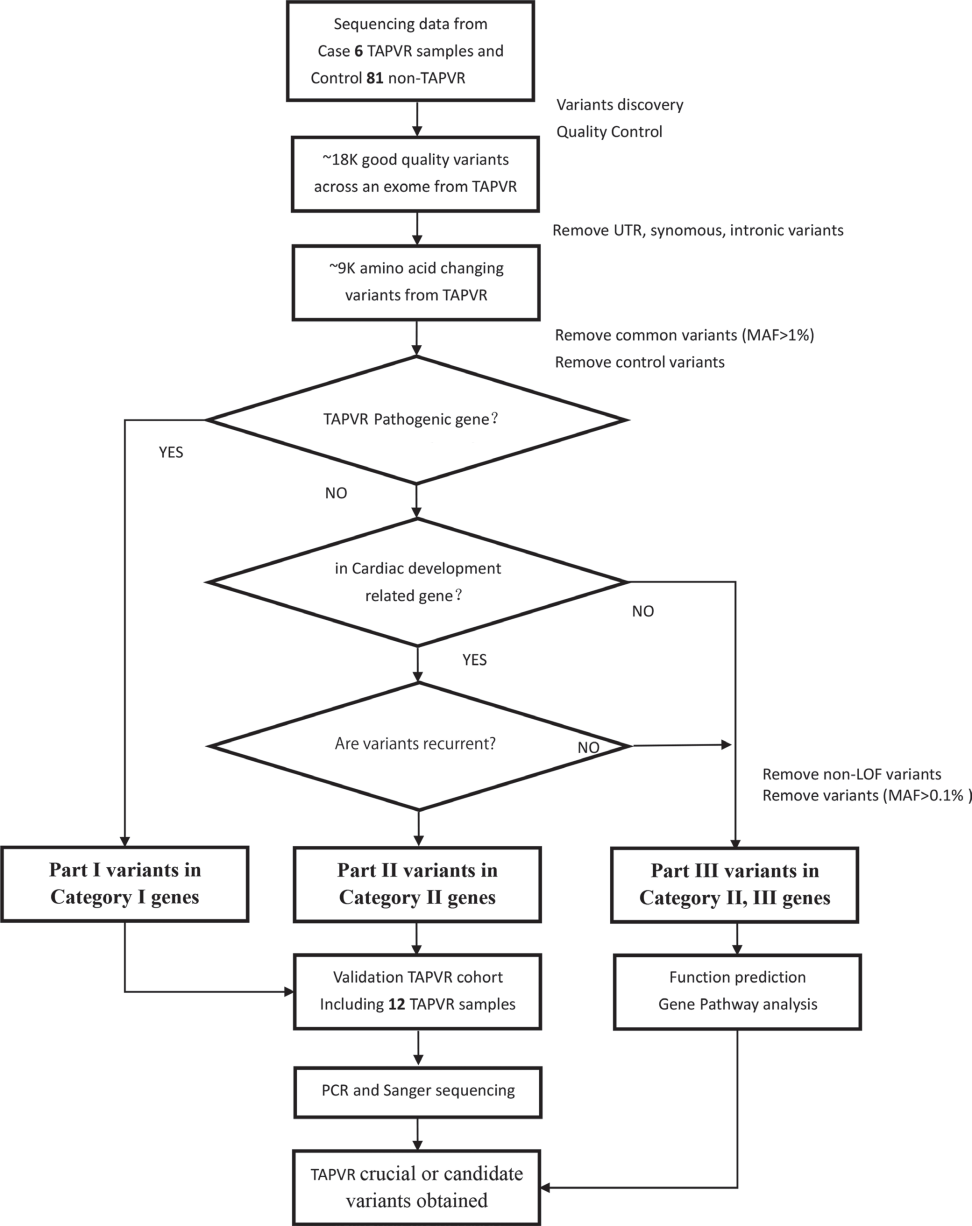
Variant filtration workflow Abbreviations are as follows: MAF, minor allele frequency; SNVs, single nucleotide variants; UTR, untranslated region.

We sequenced six sporadic TAPVR cases with mean coverage of 77-fold (~ 8.33 GB Raw data yield per individual with paired-end, 100bp reads) (Supplementary Table 5). About 19,000 (range from 18824 to 19301) primary variants with high quality were identified per individual with standard GATK-haplotype calling process. Mutation screening was carried out according to American College of Medical Genetics (ACMG) criteria guidelines: (i) UTR, synonymous, intronic variants removed; (ii) variants with minor allele frequency (MAF) < 1% from dbSNP 135, 1000 Genomes, ESP and ExAC; (iii) variants completely absent from 81 controls. After filtering, we used a combined strategy to identify potentially causal variants as follows: (i) Loss of function (LOF) variants or not; (ii) Allele frequency in the population; (iii) Homozygous or heterozygous mutations; (iv) Recurrence of the variants in different gene classification [9]. Finally, all the variants were divided into three Parts according to the possibility of pathogenic [10]. In Part I variants, we found two heterozygous missense SNVs: *ACVRL1*(NM_000020.2): c.C652T, p.R218W and *SMAD9*(NM_001127217): c.C743A, p.T248K in Patient 5; In Part II variants, *SGCD*(NM_172244): c.C717G, p.D239E was recurrent heterozygous missense variant in Patient 5, 6; In Part III variants, remaining variants were mainly referring to 62 LOF variants with MAF < 0.1% (Supplementary Table 6). (Figure 2)

### Functional prediction

**Figure 3:**
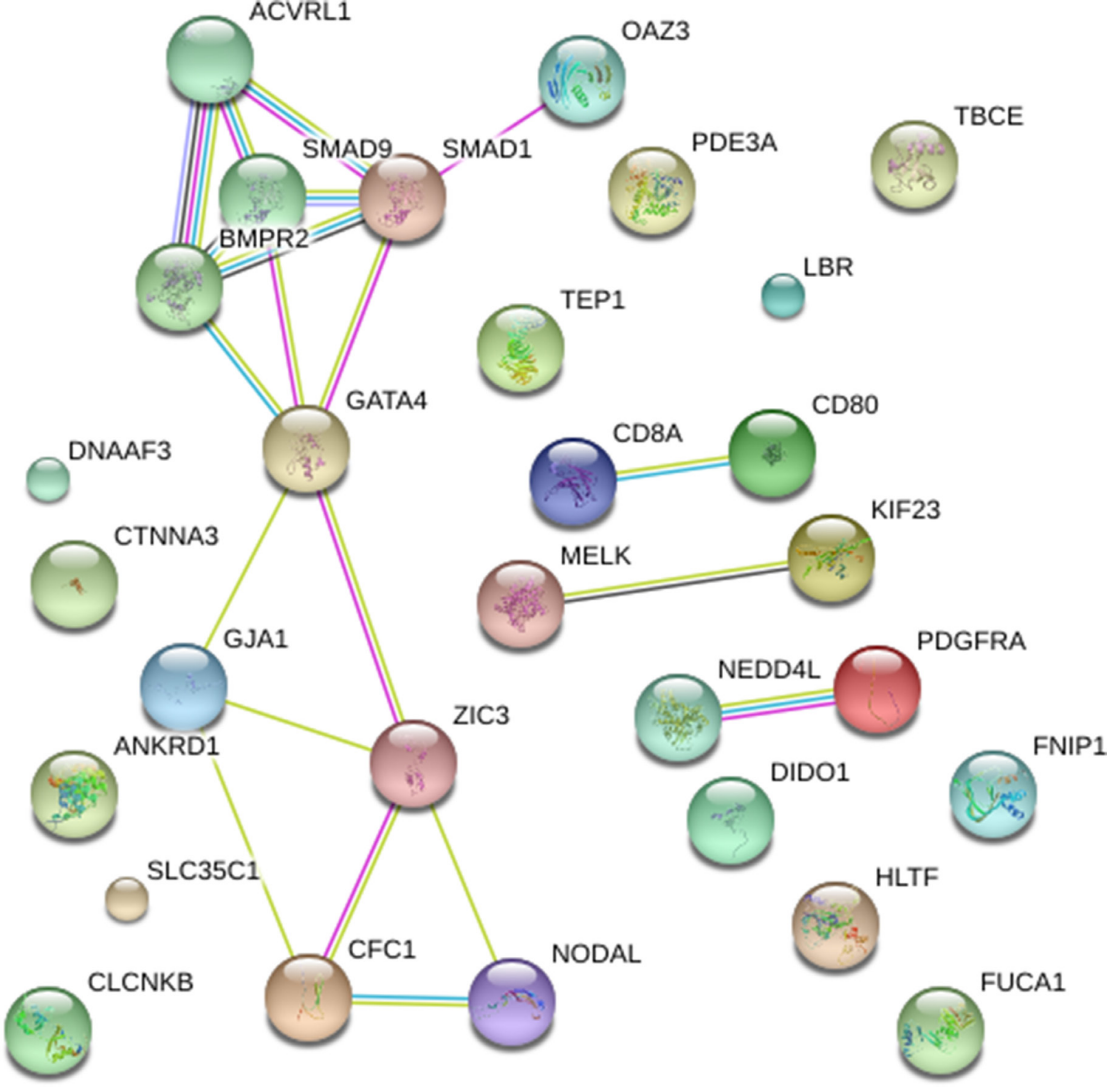
STRING pathway analysis plot (confidence score = 0.5) Network included 15 genes previously associated to TAPVR and 14 candidate genes highlighted by ToppGene analysis. The confidence view showed that *NEDD4L* associated with *PDGFRA* (combined association score = 0.819), *CD80* associated with *CD8A* (combined association score = 0.900) and *OAZ3* with *SMAD1* (combined association score = 0.566).

Firstly, mutational impacts in Part I or Part II variants were predicted from tools including SIFT, Polyphen2 and MutationTaster (Table 1). Prediction Scores of *ACVRL1*: c.C652T with high scores indicated probably or possibly damaging. However, the mutations in *SGCD* and *SMAD9* were predicted as ‘‘benign’’. Additionally, Part III variants were predicted by ToppGene (Table 2). Finally, to quantify the functional relationship, 15 genes from Category I and 14 genes from Category II and Category III genes by ToppGene *p* < 0.05 were visualized using STRING10 (Figure 3).

**Table 1:** List of selected candidate variants in Category I and Category II genes

Categories	Gene symbol	Position^a^	Chr.	Function	Ref	Alt	Protein change	SNP	ESP5400 MAF ESP	1kg2010 MAF 1000 Genomes	ExAC MAF	SIFTb	PolyPhen2c	Mutation Taster^d^
I	SMAD9	37441448	13	Nonsynonymous SNV	C	A	T248K	rs79733377	0.07%	0.86%	0.15%	No	No	No
I	ACVRL1	52308249	12	Nonsynonymous SNV	C	T	R218W	rs199874575	0.01%	0.02%	0.04%	Yes	Pb	Yes
II	SGCD	156184733	5	Nonsynonymous SNV	C	G	D239E	rs180898690	–	0.24%	0.10%	No	No	No

Abbreviations are as follows: Chr., chromosome; MAF, minor allele frequency.

^a^Human genome build 37/hg19.

^b^SIFT: Yes, damaging; No, tolerated.

^c^PolyPhen: Pb, probably damaging; Ps, possibly damaging; No, benign.

^d^MutationTaster: yes, disease causing; No, polymorphism.

**Table 2:** 14 genes of Category II and Category III genes by ToppGene p < 0.05

Rank	Gene Symbol	Gene Id	Average Score	Overall *p* Value
1	*HLTF*	6596	0.5327	0.0062
2	*CTNNA3*	29119	0.5252	0.0106
3	*CD80*	941	0.5720	0.0127
4	*DNAAF3*	352909	0.4817	0.0197
5	*NEDD4L*	23327	0.4399	0.0202
6	*MELK*	9833	0.4253	0.0216
7	*TBCE*	6905	0.3860	0.0241
8	*FNIP1*	96459	0.4714	0.0286
9	*OAZ3*	51686	0.5132	0.0289
10	*TEP1*	7011	0.4142	0.0289
11	*PDE3A*	5139	0.3717	0.0302
12	*DIDO1*	11083	0.4533	0.0353
13	*CLCNKB*	1188	0.2675	0.0390
14	*KIF23*	9493	0.2973	0.0489

63 genes were set as test genes, while 15 TAPVR Pathogenic or likely Pathogenic genes were set as training genes. The results showed 14 genes were highly correlated with TAPVR, indicating that the SNVs in these genes may be deleterious.

### Validation of variants

**Figure 4:**
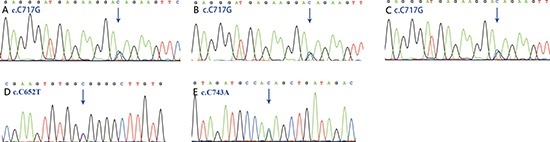
Sanger chromatograms of mutations The mutation in *SGCD* (c.C717G) was detected in 2 discovery cases (**A**, **B**) and 1validation cases (**C**). The mutations in *ACVRL1* (c.C652T) *and SMAD9* (c.C743A) were also replicated by Sanger re-sequencing (**D, E**).

In the validation stage, the identified exome sequencing candidate variants were confirmed by PCR-based Sanger sequence in 6 discovery TAPVR cases and another 12 validation TAPVR cases (Figure 4). Primers of Sanger sequencing were listed (Supplementary Table 7).

## DISCUSSION

In our study, 18 TAPVR cases and 81 non-TAPVR controls were included to reveal the genetic etiology of TAPVR in Chinese population. We screened out candidate pathogenic variants in 6 TAPVR patients, and replicated these variants in another 12 TAPVR cases. Further results were annotated and classified into several ranks based on the functional impacts. We provided evidence for *ACVRL1* as a known causative gene and for *SGCD* as candidate genes for TAPVR. Although potential disease-driving regions or genes (e.g., 4p13-q12, *ANKRD1*, etc.) in previous studies were detected, we only found few intron or UTR variants in *ANKRD1* or *PDGFRA* in our data. It may be ethnic heterogeneity because the previous studies mainly focused on Utah family rather than Chinese family TAPVR.

A rare missense mutation in *ACVRL1* (c.C652T, p.R218W) was identified as a causal mutation for TAPVR. Activin receptor like kinase 1 (*ACVRL1*) is a type I cell-surface receptor for the TGF-beta superfamily of ligands, located on chromosome 12q13. Johnson et al. [11] noted that the high expression of *ACVRL1* in highly vascularized tissues such as lung and placenta. More than 225 mutations in *ACVRL1* have been reported in HGMD (The Human Gene Mutation Database). Notably, C652T was reported in vein of Galen aneurysmal malformation (VGAM) [12]. T649G (p.T217G), G650A (p.W217X) [13–15] and G656A (p.G219H) [16] in nearby were also critical for HHT1 or pulmonary hypertension. It is interesting that we aimed at finding new TAPVR gene, but WES ultimately led to the identification a known causal variant in a different but related disease. Pulmonary hypertension is a rare lung disorder in which the arteries that carry blood from the heart to the lungs become narrowed, making it difficult for blood to flow through the vessels. All of the TAPVR patients sequenced suffered from pulmonary hypertension and mostly recovered after surgery. Therefore, we could infer that mutations in *ACVRL1* may account for TAPVR.

A recurrent mutation in *SGCD* (c.C717G, p.D239E) was detected, which coded for the dystrophin-glycoprotein complex (*DGC*). Mutations of *SGCD* were accompanied with dilated cardiomyopathy and muscular dystrophy in Caucasians, although not previously reported on TAPVR [17, 18]. Particularly, the recent study confirmed that the c.S151A mutation in *SGCD* causes a mild, subclinical cardiomyopathy phenotype [19]. Notably, they were found in 2/6 discovery patients, 1/12 validation patients and not in 81 control samples. Therefore, C717G were proven more responsible for TAPVR.

In contrast to previous studies [5, 7], whole-exome sequencing has already arrived in the clinic and was a powerful tool to investigate candidate mutations for rare diseases. The FORGE (Finding of Rare Disease Genes) Canada Consortium studied 264 disorders from the 371 submitted and identified disease-causing variants for 146 disorders over a 2-year period [20]. Several limitations in our study should be concerned including sample size. However, considering the low incidence of TAPVAR in population, our study is still a good attempt. In addition, analysis only focused on variations in coding regions, information for other regions were missing (such as introns, UTR or intergenic regions) [21]. Taken together, our findings need to be further validated in functional studies or large well-designed population-based studies in the future.

In conclusion, our study was the first attempt to dissect the etiology of TAPVR using whole-exome sequencing strategy in Chinese population. The results will provide important value to translate mutations detected by whole-exome sequencing to clinical diagnosis.

## MATERIALS AND METHODS

### Subjects

All of the TAPVR children were recruited from Nanjing Children's Hospital, Nanjing Medical University. The discovery cohort consisted of 6 cases (mean age: 5 months (range 1–11 months); gender: 1 female, 5 male) (Supplementary Table 1). The replication cohort consisted of 12 cases (mean age: 27 months (range 1–152 months); gender: 8 female, 4 male). All patients are Non-syndromic TAPVR with atrial septal defects (ASD) or pulmonary hypertension (PH). We performed Identity-by-descent (IBD) on 6 cases using PLINK 1.07 and confirmed no blood relationship on them (Supplementary Table 2). All of patients were proved by echocardiography, 12-lead electrocardiography and surgery. An in-house control database, including 81 non-TAPVR individuals, were also performed WES. All subjects included in this study have written informed consent, and the study was approved by the institutional ethical committee of Nanjing Medical University. All methods and experimental protocols were approved by Jiangsu Key Lab of Cancer Biomarkers, Prevention and Treatment, Collaborative Innovation Center for Cancer Personalized Medicine, and carried out in accordance with the approved guidelines.

### Extraction of DNA

A DNA extraction procedure was carried out using a QIAamp™ DNA and Blood Mini kit Qiagen™) according to the manufacturer's protocols. Total DNA concentration and quantity were assessed by measuring absorbance at 260 nm with NanoDrop 2000c Spectrophotometer (Thermo Scientific™).

### Library preparation and sequencing

Genomic DNA from whole blood (0.2 μg DNA) and was captured using the Agilent SureSelect Human All Exon v5 Kit. Input amounts were fragmented to a size range of 200 bp followed by end repair, adaptor ligation, and 11 PCR cycles. Appropriate amounts of enrichment DNA libraries were barcoded, pooled and loaded to lanes of a HiSeq Flow Cell, followed by 101 bp paired-end sequencing using Illumina HiSeq 1500 platform according to manufacturer's protocol.

### Bioinformatics analysis

For whole-exome sequencing (WES), image analysis and base calling were performed with CASAVA v1.8.2 using default parameters. The sequence reads were aligned to the hg19 reference sequence using BWA [22]. Picard tools were used to mark duplicates, and then multiple GATK tools (GATK LeftAlignIndels, IndelRealligner and Base Quality score recalibration) were applied to improve alignment accuracy. Variant discovery and genotype calling of single nucleotide variants (SNVs), insertions and deletions were performed on all individuals globally using the HaplotypeCaller modules of Genome Analysis Toolkit (GATK v2.8) [23]. Variant annotation process was performed using SnpEff, SnpSift (http://snpeff.sourceforge.net) and ANNOVAR(http://annovar.openbioinformatics.org/en/latest/). The Integrative Genomics Viewer (IGV)(http://www.broadinstitute.org/igv), a high-performance visualization tool, was used to check the variations manually according to genomic position [24].

### PCR-based sanger resequencing

PCR Primers were designed for the target regions and were used to amplify these regions by PCR for Sanger resequencing. Mutations were validated according to the resulting data screened through Chromas 2.4.1 and Dnaman 6.0.

### Functional annotation tools

Widely used functional annotation tools for mutations were integrated together to evaluate the biological functions and further interactions: Transcriptome Ontology Pathway PubMed based prioritization of Genes (ToppGene, http://toppgene.cchmc.org) works by gene list enrichment analysis and candidate gene prioritization based on functional annotations and protein interactions network [25]; Search Tool for the Retrieval of Interacting Genes/Proteins (STRING, http://string.embl.de) generalizes access to protein interaction data, by integrating known and predicted interactions from a variety of sources [26].

## SUPPLEMENTARY MATERIALS FIGURES AND TABLES







## References

[1] Bleyl S, Ruttenberg HD, Carey JC, Ward K (1994). Familial total anomalous pulmonary venous return: a large Utah-Idaho family. Am J Med Genet.

[2] Correa-Villasenor A, Ferencz C, Boughman JA, Neill CA (1991). Total anomalous pulmonary venous return: familial and environmental factors. The Baltimore-Washington Infant Study Grouppart Teratology.

[3] Neill CA, Ferencz C, Sabiston DC, Sheldon H (1960). The familial occurrence of hypoplastic right lung with systemic arterial supply and venous drainage “scimitar syndrome”. Bull Johns Hopkins Hosp.

[4] Fahed AC, Gelb BD, Seidman JG, Seidman CE (2013). Genetics of congenital heart disease: the glass half empty. Circ Res.

[5] Cinquetti R, Badi I, Campione M, Bortoletto E, Chiesa G, Parolini C, Camesasca C, Russo A, Taramelli R, Acquati F (2008). Transcriptional deregulation and a missense mutation define ANKRD1 as a candidate gene for total anomalous pulmonary venous return. Hum Mutat.

[6] Bleyl S, Nelson L, Odelberg SJ, Ruttenberg HD, Otterud B, Leppert M, Ward K (1995). A gene for familial total anomalous pulmonary venous return maps to chromosome 4p13-q12. Am J Hum Genet.

[7] Bleyl SB, Saijoh Y, Bax NA, Gittenberger-de Groot AC, Wisse LJ, Chapman SC, Hunter J, Shiratori H, Hamada H, Yamada S, Shiota K, Klewer SE, Leppert MF (2010). Dysregulation of the PDGFRA gene causes inflow tract anomalies including TAPVR: integrating evidence from human genetics and model organisms. Hum Mol Genet.

[8] Nash D, Arrington CB, Kennedy BJ, Yandell M, Wu W, Zhang W, Ware S, Jorde LB, Gruber PJ, Yost HJ, Bowles NE, Bleyl SB (2015). Shared Segment Analysis and Next-Generation Sequencing Implicates the Retinoic Acid Signaling Pathway in Total Anomalous Pulmonary Venous Return (TAPVR). PLoS One.

[9] Richards S, Aziz N, Bale S, Bick D, Das S, Gastier-Foster J, Grody WW, Hegde M, Lyon E, Spector E, Voelkerding K, Rehm HL (2015). Standards and guidelines for the interpretation of sequence variants: a joint consensus recommendation of the American College of Medical Genetics and Genomics and the Association for Molecular Pathology. Genet Med.

[10] Lee H, Deignan JL, Dorrani N, Strom SP, Kantarci S, Quintero-Rivera F, Das K, Toy T, Harry B, Yourshaw M, Fox M, Fogel BL, Martinez-Agosto JA (2014). Clinical exome sequencing for genetic identification of rare Mendelian disorders. JAMA.

[11] Johnson DW, Berg JN, Baldwin MA, Gallione CJ, Marondel I, Yoon SJ, Stenzel TT, Speer M, Pericak-Vance MA, Diamond A, Guttmacher AE, Jackson CE, Attisano L (1996). Mutations in the activin receptor-like kinase 1 gene in hereditary haemorrhagic telangiectasia type 2. Nat Genet.

[12] Chida A, Shintani M, Wakamatsu H, Tsutsumi Y, Iizuk Y, Kawaguchi N, Furutani Y, Inai K, Nonoyama S, Nakanishi T (2013). ACVRL1 gene variant in a patient with vein of Galen aneurysmal malformation. Journal of Pediatric Genetics.

[13] Richards-Yutz J, Grant K, Chao EC, Walther SE, Ganguly A (2010). Update on molecular diagnosis of hereditary hemorrhagic telangiectasia. Hum Genet.

[14] Olivieri C, Pagella F, Semino L, Lanzarini L, Valacca C, Pilotto A, Corno S, Scappaticci S, Manfredi G, Buscarini E, Danesino C (2007). Analysis of ENG and ACVRL1 genes in 137 HHT Italian families identifies 76 different mutations (24 novel) Comparison with other European studies. J Hum Genet.

[15] Bossler AD, Richards J, George C, Godmilow L, Ganguly A (2006). Novel mutations in ENG and ACVRL1 identified in a series of 200 individuals undergoing clinical genetic testing for hereditary hemorrhagic telangiectasia (HHT): correlation of genotype with phenotype. Hum Mutat.

[16] Lenato GM, Lastella P, Di Giacomo MC, Resta N, Suppressa P, Pasculli G, Sabba C, Guanti G (2006). DHPLC-based mutation analysis of ENG and ALK-1 genes in HHT Italian population. Hum Mutat.

[17] Tsubata S, Bowles KR, Vatta M, Zintz C, Titus J, Muhonen L, Bowles NE, Towbin JA (2000). Mutations in the human delta-sarcoglycan gene in familial and sporadic dilated cardiomyopathy. J Clin Invest.

[18] Bauer R, Hudson J, Muller HD, Sommer C, Dekomien G, Bourke J, Routledge D, Bushby K, Klepper J, Straub V (2009). Does delta-sarcoglycan-associated autosomal-dominant cardiomyopathy exist?. Eur J Hum Genet.

[19] Rutschow D, Bauer R, Gohringer C, Bekeredjian R, Schinkel S, Straub V, Koenen M, Weichenhan D, Katus HA, Muller OJ (2014). S151A delta-sarcoglycan mutation causes a mild phenotype of cardiomyopathy in mice. Eur J Hum Genet.

[20] Beaulieu CL, Majewski J, Schwartzentruber J, Samuels ME, Fernandez BA, Bernier FP, Brudno M, Knoppers B, Marcadier J, Dyment D, Adam S, Bulman DE, Jones SJ (2014). FORGE Canada Consortium: outcomes of a 2-year national rare-disease gene-discovery project. Am J Hum Genet.

[21] Jia Y, Louw JJ, Breckpot J, Callewaert B, Barrea C, Sznajer Y, Gewillig M, Souche E, Dehaspe L, Vermeesch JR, Lambrechts D, Devriendt K, Corveleyn A (2015). The diagnostic value of next generation sequencing in familial nonsyndromic congenital heart defects. Am J Med Genet A.

[22] Li H, Durbin R (2010). Fast and accurate long-read alignment with Burrows-Wheeler transform. Bioinformatics.

[23] DePristo MA, Banks E, Poplin R, Garimella KV, Maguire JR, Hartl C, Philippakis AA, del Angel G, Rivas MA, Hanna M, McKenna A, Fennell TJ, Kernytsky AM (2011). A framework for variation discovery and genotyping using next-generation DNA sequencing data. Nat Genet.

[24] Thorvaldsdottir H, Robinson JT, Mesirov JP (2013). Integrative Genomics Viewer (IGV): high-performance genomics data visualization and exploration. Brief Bioinform.

[25] Chen J, Xu H, Aronow BJ, Jegga AG (2007). Improved human disease candidate gene prioritization using mouse phenotype. BMC Bioinformatics.

[26] von Mering C, Jensen LJ, Kuhn M, Chaffron S, Doerks T, Kruger B, Snel B, Bork P (2007). STRING 7--recent developments in the integration and prediction of protein interactions. Nucleic Acids Res.

