# Supplementary material for: Whole-exome sequencing identifies SGCD and ACVRL1 mutations associated with total anomalous pulmonary venous return (TAPVR) in Chinese population
Source: Oncotarget. 2017 Feb 17;8(17):27812–9. doi: 10.18632/oncotarget.15434 (PMC5438610; doi:10.18632/oncotarget.15434)
Supplement: Supplementary file 1 [file oncotarget-08-27812-s001.pdf]

Whole-exome sequencing identifies *SGCD* and *ACVRL1* mutations associated with total anomalous pulmonary venous return (TAPVR) in Chinese population

Supplementary Materials

Supplementary Table 1: List of 6 TAPVR discovery cohort about clinical information

Patient ID	Age	Sex	Diagnosis
Patient 1	11 months 16 days	Male	TAPVR/ASD/PH
Patient 2	3 months 11 days	Male	TAPVR/ASD/PH
Patient 3	1 month 13 days	Male	TAPVR/ASD/PH
Patient 4	2 months 10 days	Female	TAPVR/ASD/PH
Patient 5	8 months 14 days	Male	TAPVR/ASD/PH
Patient 6	4 months 8 days	Male	TAPVR/ASD/PH

Abbreviations are as follows: TAPVR: Total anomalous pulmonary venous return; ASD: Atrial septal defect; PH: Pulmonary hypertension.

Supplementary Table 2: Identity-by-descent (IBD) estimation on 6 TAPVR cases using PLINK 1.07

FID1	FID2	Z0	Z1	Z2	PI_HAT	PHE	DST	PPC	RATIO
Patient 1	Patient 2	0.6584	0.2777	0.0639	0.2027	–1	0.761398	1	2.5340
Patient 1	Patient 3	0.6789	0.2558	0.0654	0.1932	–1	0.759370	1	2.4901
Patient 1	Patient 4	0.7103	0.2050	0.0847	0.1872	–1	0.759250	1	2.5930
Patient 1	Patient 5	0.7110	0.2195	0.0695	0.1793	–1	0.756506	1	2.6233
Patient 1	Patient 6	0.7072	0.2174	0.0755	0.1841	–1	0.757974	1	2.7923
Patient 2	Patient 3	0.6865	0.2368	0.0767	0.1951	–1	0.760512	1	2.4980
Patient 2	Patient 4	0.6982	0.2242	0.0776	0.1897	–1	0.759365	1	2.4273
Patient 2	Patient 5	0.7284	0.2081	0.0635	0.1676	–1	0.753497	1	2.3861
Patient 2	Patient 6	0.6964	0.2344	0.0692	0.1864	–1	0.758076	1	2.5244
Patient 3	Patient 4	0.7645	0.1342	0.1013	0.1684	–1	0.756086	1	2.3814
Patient 3	Patient 5	0.7574	0.1516	0.0910	0.1668	–1	0.755078	1	2.4064
Patient 3	Patient 6	0.7540	0.1668	0.0792	0.1626	–1	0.753373	1	2.3980
Patient 4	Patient 5	0.6285	0.3180	0.0535	0.2125	–1	0.762932	1	2.8567
Patient 4	Patient 6	0.6875	0.2432	0.0693	0.1909	–1	0.759087	1	2.5954
Patient 5	Patient 6	0.6742	0.2550	0.0708	0.1983	–1	0.760857	1	2.7334

Abbreviations are as follows: FID1: Family ID for first individual; FID2: Family ID for second individual; Z0: P(IBD = 0); Z1: P(IBD = 1); Z2: P(IBD = 2); PI_HAT: P(IBD = 2) + 0.5*P(IBD = 1) (proportion IBD); PHE: Pairwise phenotypic code (1,0,-1 = AA, AU and UU pairs); DST: IBS distance (IBS2 + 0.5*IBS1) / (N SNP pairs); PPC: IBS binomial test; RATIO: Of HETHET: IBS 0 SNPs (expected value is 2).

Supplementary Table 3: 15 TAPVR Pathogenic or Likely Pathogenic genes

Gene ID	Gene
94	*ACVRL1*
659	*BMPR2*
925	*CD8A*
2517	*FUCA1*
2626	*GATA4*
2697	*GJA1*
3930	*LBR*
4086	*SMAD1*
4093	*SMAD9*
4838	*NODAL*
5156	*PDGFRA*
7547	*ZIC3*
27063	*ANKRD1*
55343	*SLC35C1*
55997	*CFC1*

Supplementary Table 4: 221 human cardiac development related genes from GO database. See Supplementary_Table_4

Supplementary Table 5: Summary of original exome sequencing data

Data	Patient 1	Patient 2	Patient 3	Patient 4	Patient 5	Patient 6	Mean
Raw data yield (Gb)	7.82	8.25	7.85	9.37	8.24	8.46	8.33
Total reads (M)	83.80	88.36	83.87	104.46	88.37	90.43	89.88
Mapped reads (M)	83.39	87.98	83.47	103.91	87.90	90.00	89.44
Mean depth (fold)	73.96	77.57	74.67	87.05	76.08	77.39	77.79
Coverage (%)	99.51	99.57	99.53	99.48	99.47	99.50	99.51
Average read length (bp)	100	100	100	100	100	100	100

Supplementary Table 6: 62 LOF variants, Frenquencies of these mutations were < 0.1% in the 1000 Genomes Project, ESP and ExAC. See Supplementary_Table_6

Supplementary Table 7: Mutation analysis primers

Name	Primer sequence 5′–3′
SMAD9 ex F	5′- CAAAAGTCGGAGGGAAAATG -3′
SMAD9 ex R	5′- CAAAACAGCAGGCCAGTACA -3′
ACVRL1 ex F	5′- GCCAGGGCTAGGTTCTTCTT -3′
ACVRL1 ex R	5′- AGGTGGGCTGAGAGTCCTTC -3′
SGCD ex F	5′- CAATGCAGAAGCTGGCAATA -3′
SGCD ex R	5′- AAACCTGCTTGGCTCCTTTT -3′
