# Supplementary material for: Whole-exome sequencing identifies SGCD and ACVRL1 mutations associated with total anomalous pulmonary venous return (TAPVR) in Chinese population
Source: Oncotarget. 2017 Feb 17;8(17):27812–9. doi: 10.18632/oncotarget.15434 (PMC5438610; doi:10.18632/oncotarget.15434)
Supplement: Supplementary file 2 [file oncotarget-08-27812-s002.docx]

**Supplementary Table 4: 221 human cardiac development related genes from GO database**

| UniProtKB | Gene |
| --- | --- |
| E7EPN8 | *TNNT2* |
| E9PCL8 | *SLC8A1* |
| E7EP60 | *ITGA4* |
| U3KQQ9 | *TBX18* |
| U3KPY6 | *PROX1* |
| A8MZG1 | *THBS1* |
| E9PAW7 | *TGFBR3* |
| H0YNI2 | *MEF2A* |
| F8WF07 | *MOSPD3* |
| C9JW28 | *ACVR1* |
| B4DY26 | *TGFBR1* |
| H7BZL1 | *MAP2K5* |
| F5H1T8 | *TDGF1* |
| F5H1T4 | *ERBB2* |
| E5RFK8 | *LUZP1* |
| E5RFI6 | *HDAC2* |
| A2RRA7 | *NIPBL* |
| E7EUD1 | *PPARG* |
| F5H0Z3 | *LRP6* |
| E7ETB7 | *CASP8* |
| E7ET52 | *ZFPM2* |
| C9JXR7 | *CASP3* |
| D6RF42 | *GAB1* |
| D6REQ3 | *SMAD1* |
| D6REM7 | *FGFRL1* |
| D6RDX0 | *PDGFRA* |
| I3L3L3 | *GAA* |
| J3QLR9 | *RPGRIP1L* |
| H0YMP2 | *SMAD3* |
| H0YM91 | *T* |
| H0YLW3 | *BMP4* |
| E9PQW6 | *ARID1A* |
| E9PQL4 | *SOX6* |
| E9PQJ2 | *ITGB1* |
| F5GY12 | *KCNJ8* |
| F5GY03 | *SPARC* |
| F5GX88 | *ENG* |
| F5GX43 | *AXIN2* |
| F5GX40 | *PRKDC* |
| E9PP20 | *CHD7* |
| H0YL42 | *TPM1* |
| H0YKL3 | *ALDH1A2* |
| C9JFF4 | *ATG7* |
| B1ALD5 | *SLC9A1* |
| G3V2G7 | *PSEN1* |
| C9JG86 | *SETD2* |
| H0YCZ6 | *TEAD1* |
| F8VZI4 | *FRS2* |
| F8VZA4 | *DUSP6* |
| Q13127 | *REST* |
| Q13023 | *AKAP6* |
| Q8N3I7 | *BBS5* |
| Q8N157 | *AHI1* |
| Q5H9P2 | *DKFZp686P0738* |
| E9PES4 | *KIF3A* |
| E7ERA5 | *MYH10* |
| E9PDR1 | *ERBB4* |
| C9JQC4 | *OXTR* |
| F8VW56 | *ERBB3* |
| C9JPI5 | *PBRM1* |
| B4DFP7 | *MTERF4* |
| B4DFN3 | *PKD2* |
| Q8WVH6 | *MB* |
| K7EJZ7 | *HDAC5* |
| K7EJX0 | *SMAD2* |
| K7EK41 | *PRKAR1A* |
| K7ENJ8 | *RAMP2* |
| K7ENG8 | *SMAD4* |
| K7EM78 | *GJC1* |
| H9KVD2 | *SCN5A* |
| K7EKF0 | *SMAD7* |
| J3KPA4 | *VEGFA* |
| K7EPP1 | *PPP1R13L* |
| Q9ULI3 | *HEG1* |
| C9J3S8 | *FHL2* |
| C9J330 | *NCOR2* |
| C9J1W4 | *HDAC9* |
| C9J205 | *FGFR1* |
| R4GND5 | *GATA4* |
| J3KSJ4 | *RARA* |
| D6RGK0 | *PTCD2* |
| D6RG14 | *MEF2C* |
| D6RFE7 | *WHSC1* |
| D6RD75 | *RPS6KA2* |
| Q9H013 | *ADAM19* |
| D6RBQ8 | *RBPJ* |
| B0QYX2 | *PPARA* |
| Q5VYX0 | *RNLS* |
| B0QY54 | *CBY1* |
| Q5T187 | *SHC1* |
| I3L0U1 | *MKL2* |
| D6RAS8 | *IRX4* |
| E9PRG7 | *ATM* |
| F8VXL8 | *PTCH1* |
| C9JQJ2 | *TTN* |
| H7C161 | *IFT172* |
| F8VU08 | *HHEX* |
| Q6N065 | *DKFZp686O15128* |
| C9K0P9 | *DVL3* |
| G3V4R7 | *TRIP11* |
| H0Y973 | *BBS7* |
| G3V4K1 | *NFATC4* |
| G3V4F8 | *TNNI1* |
| Q1PSW9 | *GLI2* |
| B2ZWH1 | *CACYBP* |
| C9JH98 | *NRP2* |
| M0R290 | *TEAD2* |
| E9PK75 | *RXRB* |
| F5H3C5 | *SOD2* |
| E5RH63 | *RB1CC1* |
| Q5BKY1 | *LRRC10* |
| H3BSM9 | *SALL1* |
| F5H4S8 | *NFATC1* |
| E9PL83 | *ADM* |
| E5RI60 | *ADAMTS1* |
| F6SRW8 | *IFT88* |
| E5RIU6 | *CDK1* |
| Q5D0K5 | *DICER1* |
| K7EP94 | *NF1* |
| Q9UED3 | *RALDH2* |
| Q5SUY4 | *DHRS3* |
| P52799 | *EFNB2* |
| Q01860 | *POU5F1* |
| Q9BRJ9 | *MESP1* |
| Q8N907 | *DAND5* |
| Q13326 | *SGCG* |
| Q9BQ70 | *TCF25* |
| P42892 | *ECE1* |
| O76041 | *NEBL* |
| Q8IW40 | *CCDC103* |
| Q32MK0 | *MYLK3* |
| Q9H6I2 | *SOX17* |
| Q9NPF8 | *ADAP2* |
| O60565 | *GREM1* |
| O43435 | *TBX1* |
| Q9UMR3 | *TBX20* |
| Q9UFE4 | *CCDC39* |
| P80723 | *BASP1* |
| O60902 | *SHOX2* |
| Q9HBG6 | *IFT122* |
| P57059 | *SIK1* |
| Q9H2W2 | *MIXL1* |
| P20827 | *EFNA1* |
| Q12952 | *FOXL1* |
| Q12948 | *FOXC1* |
| Q12946 | *FOXF1* |
| Q8TAX0 | *OSR1* |
| Q6W2J9 | *BCOR* |
| Q13207 | *TBX2* |
| O43623 | *SNAI2* |
| P61296 | *HAND2* |
| Q99835 | *SMO* |
| P61371 | *ISL1* |
| P61328 | *FGF12* |
| Q9H1R3 | *MYLK2* |
| A1X283 | *SH3PXD2B* |
| Q9UIG0 | *BAZ1B* |
| P98161 | *PKD1* |
| O95863 | *SNAI1* |
| P50238 | *CRIP1* |
| P50461 | *CSRP3* |
| Q8IZQ8 | *MYOCD* |
| Q92819 | *HAS2* |
| Q92629 | *SGCD* |
| Q15672 | *TWIST1* |
| O75147 | *OBSL1* |
| P48436 | *SOX9* |
| Q5T481 | *RBM20* |
| Q06945 | *SOX4* |
| O96004 | *HAND1* |
| O75610 | *LEFTY1* |
| P12883 | *MYH7* |
| P36382 | *GJA5* |
| O60481 | *ZIC3* |
| A6NCS4 | *NKX2-6* |
| O60344 | *ECE2* |
| Q0ZGT2 | *NEXN* |
| Q15468 | *STIL* |
| Q15465 | *SHH* |
| P19544 | *WT1* |
| Q8IWY4 | *SCUBE1* |
| P0DMC3 | *APELA* |
| O15350 | *TP73* |
| Q96HF1 | *SFRP2* |
| Q96HD1 | *CRELD1* |
| P35716 | *SOX11* |
| P35713 | *SOX18* |
| Q7Z494 | *NPHP3* |
| Q14863 | *POU6F1* |
| Q14814 | *MEF2D* |
| Q96NZ1 | *FOXN4* |
| Q14623 | *IHH* |
| Q96T68 | *SETDB2* |
| Q96RK4 | *BBS4* |
| Q96LD1 | *SGCZ* |
| Q96L96 | *ALPK3* |
| P30626 | *SRI* |
| P54792 | *DVL1P1* |
| P54760 | *EPHB4* |
| O94851 | *MICAL2* |
| Q99967 | *CITED2* |
| Q99959 | *PKP2* |
| Q99958 | *FOXC2* |
| Q99697 | *PITX2* |
| P11831 | *SRF* |
| Q7Z7M0 | *MEGF8* |
| Q4G0X9 | *CCDC40* |
| Q96FH0 | *MEF2BNB* |
| P52952 | *NKX2-5* |
| Q7RTU7 | *SCX* |
| Q8NEP3 | *DNAAF1* |
| Q8NDZ4 | *C3orf58* |
| Q13765 | *NACA* |
| A9UJP0 | *PDGFB* |
| A4UGR9 | *XIRP2* |
| C9IYY1 | *FOXP1* |
| C9IZ65 | *CTNNB1* |
| Q5JYX0 | *CDC42* |
| C9J9N4 | *GLI3* |
| C9J685 | *RTN4* |
| Q5TF93 | *HEY2* |
