# Supplementary material for: Whole-exome sequencing identifies SGCD and ACVRL1 mutations associated with total anomalous pulmonary venous return (TAPVR) in Chinese population
Source: Oncotarget. 2017 Feb 17;8(17):27812–9. doi: 10.18632/oncotarget.15434 (PMC5438610; doi:10.18632/oncotarget.15434)
Supplement: Supplementary file 3 [file oncotarget-08-27812-s003.docx]

**Supplementary Table 6: 62 LOF variants, Frenquencies of these mutations were <0.1% in the 1000 Genomes Project, ESP and ExAC**

| Chr | Start | End | Ref | Alt | Gene symbol | Transcript | Protein change | Function | ESP5400 MAF | 1KG2010 MAF | ExAC MAF |
| --- | --- | --- | --- | --- | --- | --- | --- | --- | --- | --- | --- |
| 1 | 16375582 | 16375584 | GA | G | *CLCNKB* | NM_001165945.2 | R40 | frameshift | 0.0004 |  | 0.0007 |
| 1 | 55223703 | 55223704 | A | AG | *PARS2* | NM_152268.3 | P377P? | frameshift |  |  | 0 |
| 1 | 85036305 | 85036306 | A | AT | *CTBS* | NM_004388.2 | M92N? | frameshift |  |  | 0 |
| 1 | 85598545 | 85598546 | T | A | *WDR63* | NM_145172.4 | Y847* | stop_gained |  |  |  |
| 1 | 145457571 | 145457572 | G | A | *POLR3GL* | NM_032305.1 | R120* | stop_gained |  |  |  |
| 1 | 150246524 | 150246529 | GATGA | G | *C1orf54* | NM_001301039.1 | DE28 | frameshift |  |  |  |
| 1 | 151739647 | 151739648 | T | C | *OAZ3* | NM_016178.2 | *59S | stop_lost |  |  |  |
| 1 | 158325616 | 158325617 | T | A | *CD1E* | NM_001185107.1 | L119* | stop_gained |  |  |  |
| 1 | 206566999 | 206567000 | C | G | *SRGAP2B* | NM_001271870.1 | Y180* | stop_gained |  | 0.0002 | 0 |
| 1 | 215781399 | 215781400 | C | T | *KCTD3* | NM_016121.3 | R451* | stop_gained |  |  | 0 |
| 1 | 235564858 | 235564861 | AAG | A | *TBCE* | NM_001287801.1 | K48 | frameshift |  |  | 0 |
| 1 | 248617053 | 248617054 | C | CTG | *OR2T2* | NM_001004136.1 | T319T? | frameshift |  |  | 0.0009 |
| 2 | 1371244 | 1371245 | A | T | *SNTG2* | NM_018968.3 | *540L | stop_lost |  |  |  |
| 2 | 170411710 | 170411712 | CA | C | *FASTKD1* | NM_024622.4 | L379 | frameshift |  |  |  |
| 2 | 202712192 | 202712193 | G | A | *CDK15* | NM_001261435.1 | W314* | stop_gained |  |  | 0 |
| 3 | 108072397 | 108072398 | G | A | *HHLA2* | NM_001282556.1 | W63* | stop_gained |  |  |  |
| 3 | 119276533 | 119276534 | A | T | *CD80* | NM_005191.3 | C14* | stop_gained |  |  |  |
| 3 | 122679993 | 122679995 | CA | C | *SEMA5B* | NM_001256347.1 | L93 | frameshift |  |  |  |
| 3 | 130380797 | 130380799 | GA | G | *COL6A6* | NM_001102608.1 | E2050 | frameshift |  |  | 0 |
| 3 | 148793803 | 148793804 | G | A | *HLTF* | NM_003071.3 | R87* | stop_gained | 0.0001 |  | 0.0001 |
| 4 | 186357226 | 186357227 | A | AAT | *C4orf47* | NM_001114357.1 | I117I? | frameshift |  |  |  |
| 5 | 61876633 | 61876634 | C | T | *LRRC70* | NM_181506.4 | R457* | stop_gained |  |  |  |
| 5 | 131080348 | 131080349 | G | A | *FNIP1* | NM_133372.2 | R43* | stop_gained |  |  |  |
| 5 | 133745622 | 133745623 | A | AT | *CDKN2AIPNL* | NM_080656.2 | Q103Q? | frameshift |  |  | 0 |
| 5 | 140214833 | 140214834 | T | TA | *PCDHA7* | NM_018910.2 | I289I? | frameshift |  |  | 0 |
| 5 | 162918134 | 162918135 | C | T | *HMMR* | NM_001142556.1 | R715* | stop_gained |  |  | 0 |
| 5 | 170239114 | 170239116 | GT | G | *GABRP* | NM_014211.2 | F393 | frameshift |  |  |  |
| 6 | 27223092 | 27223094 | GA | G | *PRSS16* | NM_005865.3 | *515 | frameshift |  |  | 0 |
| 6 | 99887704 | 99887710 | GTTTAC | G | *USP45* | NM_001080481.1 | VN699 | frameshift |  |  | 0 |
| 6 | 137468890 | 137468891 | G | A | *IL22RA2* | NM_052962.2 | R204* | stop_gained | 0.0001 | 0.0004 | 0.0002 |
| 7 | 134852510 | 134852511 | A | AAT | *C7orf49* | NM_024033.3 | V62V? | frameshift |  |  |  |
| 7 | 142881508 | 142881511 | CAA | C | *TAS2R39* | NM_176881.2 | PK333 | frameshift |  |  | 0.0001 |
| 7 | 150554652 | 150554654 | AG | A | *AOC1* | NM_001272072.1 | G366 | frameshift |  |  | 0 |
| 8 | 95172359 | 95172362 | TTG | T | *CDH17* | NM_001144663.1 | T463 | frameshift |  |  |  |
| 9 | 36657248 | 36657249 | G | A | *MELK* | NM_014791.3 | W355* | stop_gained |  |  |  |
| 10 | 67748465 | 67748467 | CG | C | *CTNNA3* | NM_001127384.2 | R750 | frameshift |  |  |  |
| 11 | 49053362 | 49053364 | AG | A | *TRIM49B* | NM_001206626.1 | K71 | frameshift |  |  |  |
| 11 | 55595522 | 55595528 | TTCTAC | T | *OR5L2* | NM_001004739.1 | FY277 | frameshift |  |  | 0 |
| 11 | 64360909 | 64360910 | C | A | *SLC22A12* | NM_144585.3 | Y180* | stop_gained |  |  |  |
| 11 | 101786090 | 101786091 | G | GC | *KIAA1377* | NM_020802.3 | A26A? | frameshift | 0.0001 |  | 0 |
| 12 | 6657924 | 6657925 | C | A | *IFFO1* | NM_001193457.1 | E391* | stop_gained |  |  |  |
| 12 | 20769301 | 20769302 | G | T | *PDE3A* | NM_000921.4 | E470* | stop_gained |  |  |  |
| 13 | 49281741 | 49281742 | T | TC | *CYSLTR2* | NM_020377.2 | H264P? | frameshift |  |  |  |
| 14 | 20844322 | 20844324 | AG | A | *TEP1* | NM_007110.4 | P2063 | frameshift |  |  | 0.0003 |
| 14 | 57082702 | 57082703 | T | TC | *TMEM260* | NM_017799.3 | I300I? | frameshift |  |  |  |
| 15 | 69728928 | 69728931 | CAG | C | *KIF23* | NM_138555.3 | Q475 | frameshift |  |  |  |
| 16 | 10524917 | 10524922 | TCAGA | T | *ATF7IP2* | NM_024997.3 | QT148 | frameshift |  |  |  |
| 16 | 23702332 | 23702334 | AC | A | *ERN2* | NM_033266.3 | V915 | frameshift |  |  | 0 |
| 16 | 67384108 | 67384110 | AG | A | *LRRC36* | NM_018296.5 | R165 | frameshift |  |  |  |
| 16 | 72142193 | 72142194 | C | G | *DHX38* | NM_014003.3 | Y1011* | stop_gained |  |  |  |
| 17 | 40945696 | 40945699 | AAG | A | *WNK4* | NM_032387.4 | K749 | frameshift |  |  |  |
| 17 | 43220911 | 43220912 | C | T | *ACBD4* | NM_001135705.1 | R298* | stop_gained |  |  | 0 |
| 17 | 67189671 | 67189674 | TTC | T | *ABCA10* | NM_080282.3 | R534 | frameshift |  |  |  |
| 17 | 76167884 | 76167885 | C | G | *SYNGR2* | NM_004710.3 | Y181* | stop_gained |  |  |  |
| 18 | 29340291 | 29340293 | CT | C | *SLC25A52* | NM_001034172.2 | K121 | frameshift |  |  |  |
| 18 | 56010230 | 56010231 | C | T | *NEDD4L* | NM_001144967.2 | Q491* | stop_gained |  |  |  |
| 19 | 33247946 | 33247947 | C | G | *TDRD12* | NM_001110822.1 | S259* | stop_gained |  |  |  |
| 19 | 55677734 | 55677735 | C | T | *DNAAF3* | NM_001256714.1 | W63* | stop_gained |  |  |  |
| 20 | 2796398 | 2796400 | TC | T | *C20orf141* | NM_001256538.1 | F159 | frameshift |  |  |  |
| 20 | 2797386 | 2797388 | TG | T | *TMEM239* | NM_001167670.1 | W63 | frameshift |  |  |  |
| 20 | 34091009 | 34091010 | C | T | *CEP250* | NM_007186.4 | Q1605* | stop_gained |  |  |  |
| 20 | 61537229 | 61537234 | GATAC | G | *DIDO1* | NM_080796.3 | MY531 | frameshift |  |  |  |
| 21 | 31803030 | 31803031 | T | A | *KRTAP13-4* | NM_181600.1 | C146* | stop_gained |  | 0.0002 | 0 |
